# A rare case of combined thymoma and a multilocular thymic cyst discovered due to chest pain

**DOI:** 10.1186/s40792-021-01243-2

**Published:** 2021-07-08

**Authors:** Kengo Yasuda, Yoshiteru Kidokoro, Karen Makishima, Shinji Matsui, Atsuyuki Nakanishi, Yuji Nozaka, Yuki Oshima, Yasuaki Kubouchi, Yuzo Takagi, Tomohiro Haruki, Hiroshige Nakamura

**Affiliations:** 1 Center for Clinical Residency Program, Tottori University Hospital, 86, Nishicho, Yonago, Tottori, 683-8503 Japan; 2 Division of General Thoracic Surgery and Breast and Endocrine Surgery, Department of Surgery, Faculty of Medicine, Tottori University, 86, Nishicho, Yonago, Tottori, 683-8503 Japan; 3 Department of Pathology, Faculty of Medicine, Tottori University, 86, Nishicho, Yonago, Tottori, 683-8503 Japan

**Keywords:** Thymoma, Chest pain, MTC

## Abstract

**Background:**

A thymoma with chest pain and multilocular thymic cysts (MTCs) is very rare.

**Case presentation:**

A 49-year-old man presented to another hospital complaining of an anterior chest pain. Chest computed tomography (CT) showed an anterior mediastinal tumor 60 × 30 × 55 mm in size. The boundary with the pericardium or left brachiocephalic vein seemed to be partially unclear while enhanced by the contrast medium, and so the tumor could have invaded them. No definitive diagnosis of myasthenia gravis was made although the serum anti-acetylcholine receptor antibody count was high. We performed an extended thymectomy with combined partial resection of left brachiocephalic vein, left upper lobe, and left phrenic nerve. He was discharged with no chest pain and no complications post-surgery. The tumor was pathologically type B2 thymoma with hemorrhage necrosis and MTCs, and we diagnosed Masaoka stage II because of no histological infiltration to the organs.

**Conclusions:**

We speculated that hemorrhagic necrosis due to infarction in tumor caused the inflammation to spread to the surrounding organs, which was related to the chest pain and the development of MTCs.

## Background

In general, a thymoma with chest pain is rare. The cause of chest pain has been reported to be associated with the necrosis in tumor [1–3], but the cause of necrosis has not been revealed yet. In addition, a thymoma with multilocular thymic cysts (MTCs) is also a rare lesion, accounting for approximately 3% of all anterior mediastinal masses [4]. There have been few reports of thymoma with a multilocular thymic cyst discovered due to chest pain. Thus, we report a rare case of thymoma with MTCs discovered due to chest pain, referred to a review of the literatures.

## Case presentation

**Fig. 1:**
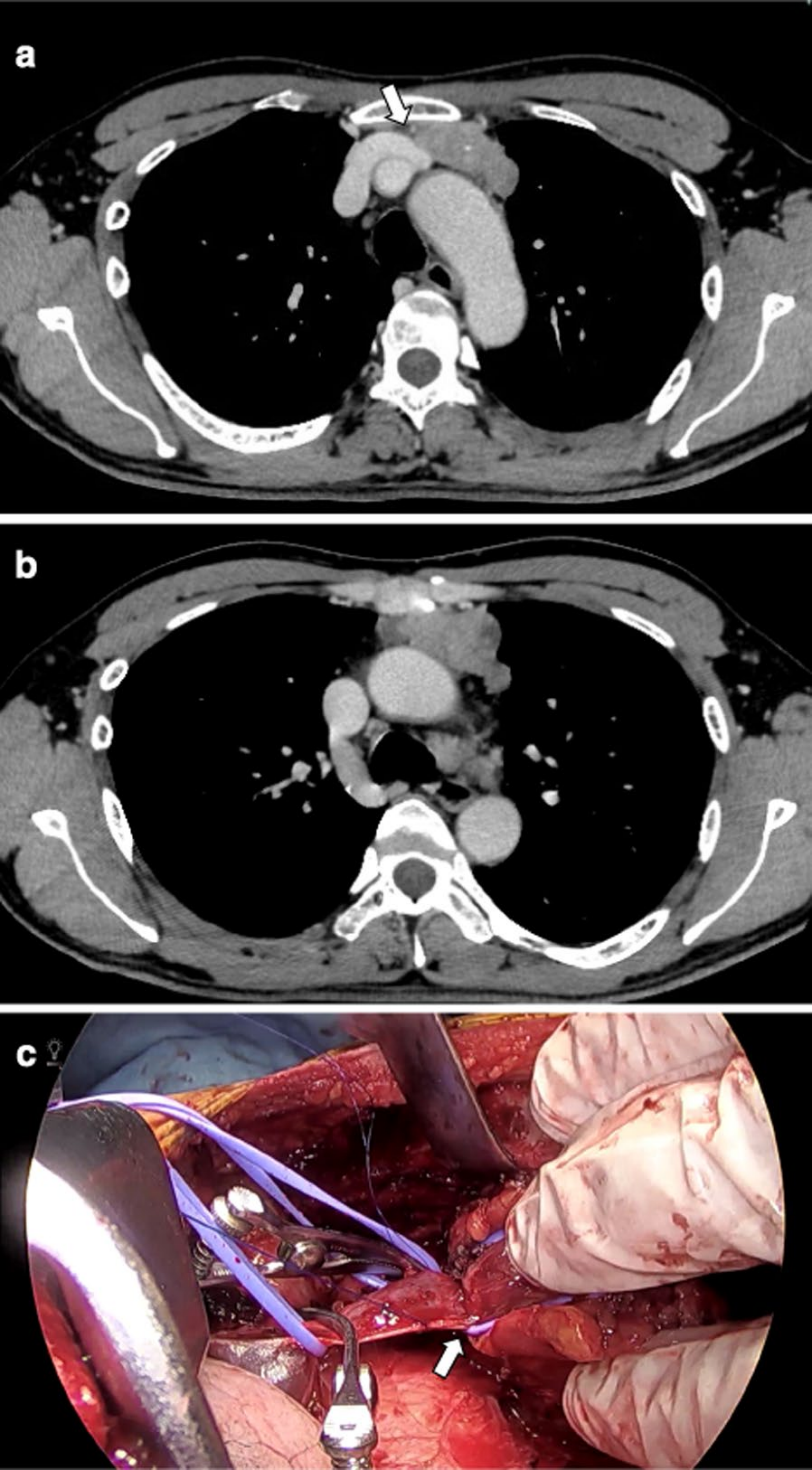
Preoperative chest computed tomography (CT) findings showed a 60 × 30 × 55 mm anterior mediastinal tumor with calcification. Contrast-enhanced CT showed mild encasement of left brachiocephalic vein (white arrow) and infiltration of the vein was suspected (**a**). The tumor contained the low-density areas and they might show necrosis or MTCs (**b**). Intraoperative findings showed strong adhesion of the left brachiocephalic vein (white arrow), and therefore partial resection of the vein was performed (**c**)

A 49-year-old man presented to another hospital complaining of an anterior chest pain. A chest computed tomography (CT) showed an anterior mediastinal mass that had cystic changes and the patient was referred to our hospital for examination and treatment. The physical examination showed no signs or symptoms of systemic or autoimmune disorder including myasthenia gravis (MG) such as muscle weakness. No definitive diagnosis of MG was made at our department of neurology. His laboratory test results revealed elevations in an anti-acetylcholine receptor antibody (AChR-ab) level (3.5 nmol/L). The contrast-enhanced CT on admission showed the size of tumor was 60 × 30 × 55 mm in diameter and the boundary with the pericardium or left brachiocephalic vein seemed to be partially unclear (Fig. 1a). CT also showed a low-density area found in a part of the tumor (Fig. 1b). The tumor was suspected to be an invasive thymoma without MG, with infiltration into the pericardium and left brachiocephalic vein, clinically Masaoka stage III.

A surgical resection was conducted through a median sternotomy. The tumor was located on the left upper portion of the thymus. It showed a strong inflammatory adhesion of the surrounding tissues of the thymus, especially the left brachiocephalic vein, left upper lobe and left phrenic nerve though there was no invasion into the pericardium. We performed an extended thymectomy combined with partial resection of left brachiocephalic vein, left upper lobe and left phrenic nerve (Fig. 1c). The total length of the operation was 246 min, and the amount of blood loss was 160 ml. The patient was discharged 8 days after the operation with no chest pain and no postoperative complications.

**Fig. 2:**
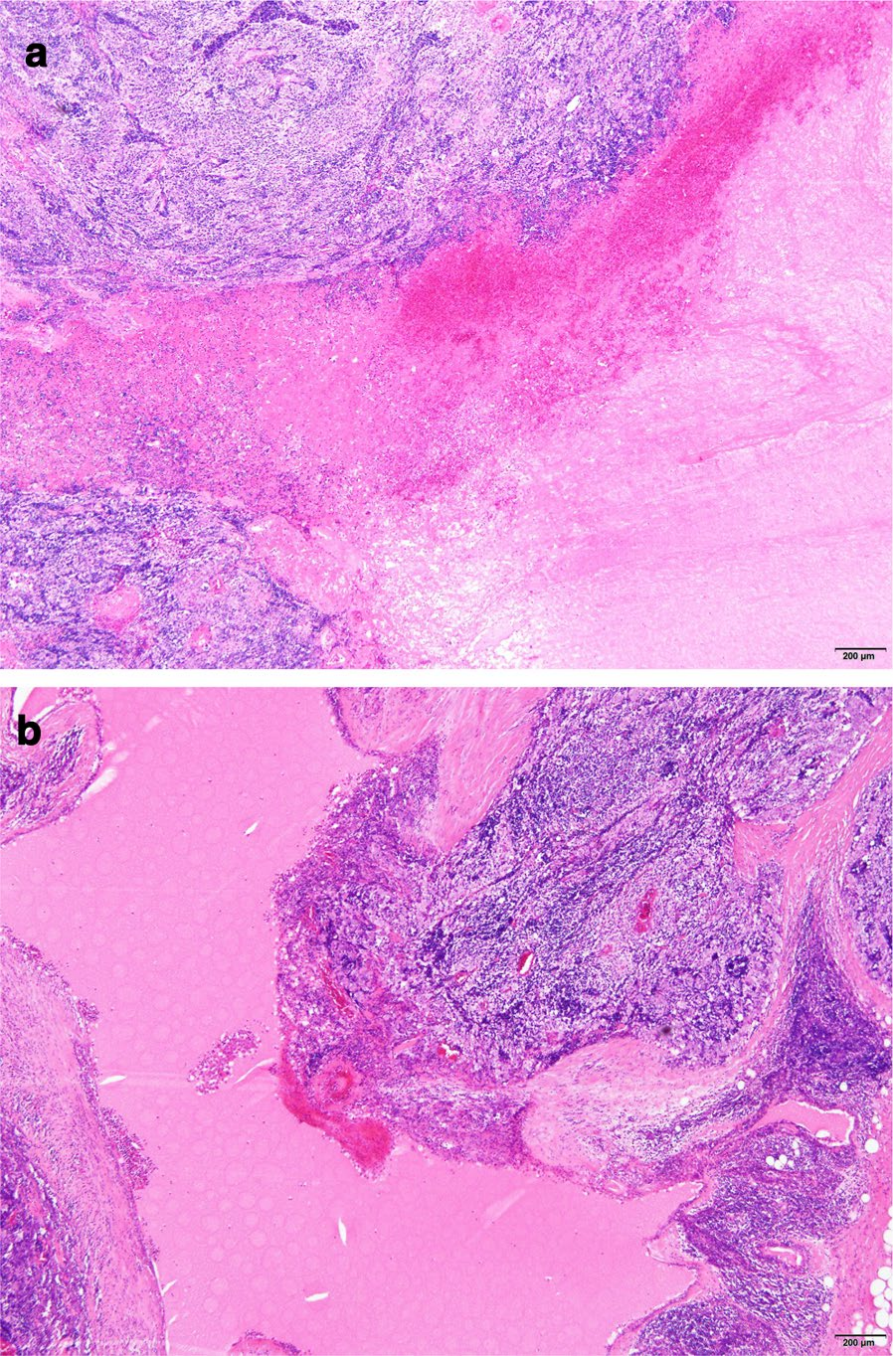
Microscopic findings showed that the tumor was 59 × 47 × 21 mm, and consisted of a solid lesion with a cystic component (hematoxylin and eosin, × 40). The tumor pathologically classified as WHO type B2 thymoma. In thymoma, there was an extensive hemorrhagic necrosis which infraction might have resulted in (**a**). There were multilocular thymic cysts combined with thymoma (**b**)

Microscopically, the operative specimen showed that the tumor was 59 × 47 × 21 mm, and consisted of a solid lesion with a cystic component. Polygonal atypical cells with eosinophilic cytoplasm and round nuclei were proliferating in sheet form. In the upper part of the tumor, there was an extensive hemorrhagic necrosis suggesting infarction (Fig. 2a). The tumor had infiltrated partially into the surrounding adipose tissue, but it did not invade the phrenic nerve, left brachiocephalic vein and left lung. Based on these findings, the mass pathologically classified as WHO type B2 and Masaoka stage II.

Microscopic findings also showed, around the thymoma, there were many cysts lined with the cubic epithelium and the atypical squamous epithelium. There was an eosinophilic reservoir including cholesterin crystals in the cysts, and Hassall corpuscles in the cystic wall, accompanied by a mild chronic inflammatory infiltration. According to these findings, we diagnosed thymoma with MTCs (Fig. 2b).

## Discussion

In general, thymomas are relatively rarely symptomatic, but there are some reports of the thymoma with chest pain. Wright et al. reported 190 patients with thymoma underwent resection, of which four cases presented clinically with chest pain and either infarction or hemorrhage (2%) [1]. There are eight cases of thymoma with chest pain reported in Japan [2, 3, 5–10] (Table 1). These reports showed adhesion with surrounding organs as intraoperative findings (six cases), pleural effusion (eight cases), and necrosis as pathological findings (seven cases). Similar to the reported cases [2, 5–7, 9, 10], in our case, intraoperative findings also showed strong adhesions to surrounding the organs. Thus, although no obvious fever or pleural effusion was observed, mediastinal and partial pleuritis could have been present and was thought to have caused the chest pain.

**Table 1:** Reported cases of thymoma with chest pain in Japanese literatures

Case	Author	Year	Age	Gender	Chest pain	Pleural effusion	Adhesion	Combined resection	Necrosis	Cysts	WHO type	Masaoka's stage
1	Ito [5]	2006	25	M	+	+	Present	RUL	+	Not listed	Type B1	I
2	Sato [6]	2010	24	M	+	+	Present	LUL	+	Pseudocysts	Type B2	II
3	Misawa [7]	2011	63	M	+	+	Present	−	+	MTCs	Type B2	II
4	Takasaki [2]	2012	46	M	+	+	Present	Pericardium	+	Not listed	Type B2	II
5	Toyokawa [8]	2014	28	M	+	+	−	−	−	MTCs	Type B2	I
6	Shiiya [3]	2015	53	M	+	+	−	−	+	Not listed	Type AB	II
7	Kataoka [9]	2015	52	F	+	+	Present	−	+	Not listed	Type B1	I
8	Yasukawa [10]	2017	71	F	+	+	Present	RUL, pericardium, brachiocephalic vein	+	Not listed	Type AB	I
9	Our case	2020	49	M	+	−	Present	LUL, phrenic nerve, brachiocephalic vein	+	MTCs	Type B2	II

*RUL* right upper lobe, *LUL* left upper lobe, *MTCs* multilocular thymic cysts

The obvious cause of the necrosis within the thymoma is still unknown. However, Wright et al. reported that thrombotic changes in the vessels near the necrosis caused an infarction and resulted in hemorrhagic necrosis in the tumor [1]. In our case, although the pathological findings showed little obvious thrombotic changes in the tumor, there was a hemorrhagic necrosis suggesting infarction probably due to an impaired vascular supply or apoptosis. The hemorrhagic necrosis suggested that the inflammation might have spread to surrounding organs.

The cases of MTCs associated with thymoma is considered to be relatively rare, Nakamura et al. reported that the prevalence of thymoma with MTCs were approximately 17% [11]. Furthermore, in Japan, there were few cases which thymoma with necrosis and MTCs (Table 1). It is speculated that MTCs are caused by an acquired inflammatory process in structures derived from the medullary duct epithelium such as Hassall corpuscles. Misawa et al. argued that the cyst ruptured and caused inflammation which resulted in chest pain [7]. On the other hand, in our case, it is probable that embolism which occurred in the thymus resulted in necrosis and chest pain. Shen et al. argued that the formation of MTCs was closely related to inflammation and there might be a histological association between MTCs and thymic neoplasms according to the results of immunohistochemical staining [11, 12]. Thus, it was possible that this embolism also caused inflammatory changes and MTCs occurred in the thymoma.

Symptoms of these thymomas were generally difficult to notice until they grew and progressed considerably. Although thymoma with chest pain adhered to surrounding organs due to the inflammation, it had a low invasion tendency and might have be detected early in a surgically resectable condition. Combined resection was required due to strong adhesion to the surrounding organs, but the postoperative pathological findings showed negative resection margins [2, 3, 5–10]. The number of cases of thymoma with chest pain or MTCs is still small, and it is necessary to accumulate cases and follow-up for a long period of time.

## Conclusions

This report presented a surgically excised case of thymoma with MTCs discovered due to chest pain. Our case indicated that these symptoms may have been caused by hemorrhagic necrosis due to infarction in tumor. Chest pain caused by inflammatory spread may develop earlier than chest pain caused by tumor infiltration, and may lead to early detection in an operable condition.

## Data Availability

Not applicable.

## Abbreviations

AchR-ab: Acetylcholine receptor antibody
CT: Computed tomography
MG: Myasthenia gravis
MTC: Multilocular thymic cyst
